# Does Respondent Driven Sampling Alter the Social Network Composition and Health-Seeking Behaviors of Illicit Drug Users Followed Prospectively?

**DOI:** 10.1371/journal.pone.0019615

**Published:** 2011-05-06

**Authors:** Abby E. Rudolph, Carl Latkin, Natalie D. Crawford, Kandice C. Jones, Crystal M. Fuller

**Affiliations:** 1 Johns Hopkins University Bloomberg School of Public Health, Baltimore, Maryland, United States of America; 2 Center for Urban Epidemiologic Studies at the New York Academy of Medicine, New York, New York, United States of America; 3 Columbia University Mailman School of Public Health, New York, New York, United States of America; University of Zaragoza, Spain

## Abstract

Respondent driven sampling (RDS) was originally developed to sample and provide peer education to injection drug users at risk for HIV. Based on the premise that drug users' social networks were maintained through sharing rituals, this peer-driven approach to disseminate educational information and reduce risk behaviors capitalizes and expands upon the norms that sustain these relationships. Compared with traditional outreach interventions, peer-driven interventions produce greater reductions in HIV risk behaviors and adoption of safer behaviors over time, however, control and intervention groups are not similarly recruited. As peer-recruitment may alter risk networks and individual risk behaviors over time, such comparison studies are unable to isolate the effect of a peer-delivered intervention. This analysis examines whether RDS recruitment (without an intervention) is associated with changes in health-seeking behaviors and network composition over 6 months. New York City drug users (N = 618) were recruited using targeted street outreach (TSO) and RDS (2006–2009). 329 non-injectors (RDS = 237; TSO = 92) completed baseline and 6-month surveys ascertaining demographic, drug use, and network characteristics. Chi-square and t-tests compared RDS- and TSO-recruited participants on changes in HIV testing and drug treatment utilization and in the proportion of drug using, sex, incarcerated and social support networks over the follow-up period. The sample was 66% male, 24% Hispanic, 69% black, 62% homeless, and the median age was 35. At baseline, the median network size was 3, 86% used crack, 70% used cocaine, 40% used heroin, and in the past 6 months 72% were tested for HIV and 46% were enrolled in drug treatment. There were no significant differences by recruitment strategy with respect to changes in health-seeking behaviors or network composition over 6 months. These findings suggest no association between RDS recruitment and changes in network composition or HIV risk, which supports prior findings from prospective HIV behavioral surveillance and intervention studies.

## Introduction

The absence of a sampling frame for marginalized and/or highly stigmatized populations (e.g., injection drug users, men who have sex with men, sex workers) makes it difficult to recruit representative samples of the target population. Respondent-driven sampling (RDS) was introduced by Heckathorn in 1997 as an approach to sampling design and inference for these “hidden” or “hard-to-reach” populations [1]. Traditional recruitment approaches use probability-based sampling methods and make inferences about the population directly from the sample. RDS is unique in that it relies on the networks of social relationships that connect members of the target population to facilitate sampling from it [2] and uses information about how members of the target population are connected to weight recruits in a way that accounts for non-random sampling.

While “respondent-driven sampling” was coined by Heckathorn, Broadhead and colleagues had been using a peer-driven approach to sample and educate injection drug users at risk for HIV since the early 1990s [3], and sociologists have used “snowball sampling” since the mid-1900s [4]. The rationale for peer driven interventions (PDIs) in HIV research is that both human immunodeficiency virus (HIV) and HIV prevention information can be transmitted through HIV risk networks (e.g., drug use and sex networks). Peer driven education interventions are a subset of peer health interventions that rely on members of the target population to both recruit and educate their peers [5]. In studies among drug users, respondents typically receive incentives for successfully recruiting other members of the target population (typically no more than 3) and for being effective peer educators or for demonstrating knowledge about HIV prevention [5], [6], [7], [8], [9], [10], [11], [12], [13], [14]. There are a variety of peer driven education interventions, some of which randomize assignment to the intervention group, and others which have been evaluated less rigorously (e.g., those comparing peer-driven interventions with traditional outreach interventions).

Peer recruitment has several advantages over traditional approaches that rely on outreach workers. First, the recruitment burden is placed on participants (in this case, active drug users) who are socially connected to other members of the target population. Because recruiters are themselves members of the target population, they can more easily identify and recruit those that may be “hidden” from traditional outreach workers who are not part of this community. Additionally, because participants recruit peers from within their own social networks, masking and volunteer biases should be reduced through social pressure exerted by their peers [1], [15], [16]. Recruitment incentives may further motivate peer recruiters to employ their social influence to successfully recruit those who are difficult to reach using conventional approaches [16]. The confidentiality of potential recruits is also protected, because those who are recruited by peers may choose to not to participate in the study and can remain anonymous to research staff. Finally, because participants are recruited by their peers, recruitment efforts are likely more culturally sensitive.

Using peers to disseminate intervention materials and/or messages is also advantageous. As drug user social networks are maintained through sharing interactions (i.e., sharing drugs, money, injection equipment, and information about drug sources, price and quality), using a peer-driven approach to disseminate educational information and to reduce risk behaviors capitalizes and expands upon the norms that sustain these relationships [12]. This intervention model often incorporates a training component (based on the principles of social learning, social identity, social norms, and social diffusion) whereby peer educators are trained to 1) serve as peer mentors, 2) effectively motivate their network members to reduce HIV risk behaviors, and 3) model safer behaviors within their network [17]. Thus, peer education and peer-driven interventions work well when HIV is transmitted through the behaviors that maintain social networks [12], [18]. For example, PDIs designed to reduce HIV risk behaviors have produced significant reductions in the number of unsafe sex practices [14] and the frequency of injection [5], [11], [12], [14], [19], [20], [21], [22], [23], [24], [25], [26], [27], [28], [29], [30], [31], [32], drug use [33], and high-risk injecting practices (e.g., sharing rinse water [5], [11], [14], cookers [5], [11], [14], filters [5], [11], [14] and syringes [5], [20], [22], [23], [24], [26], [27], [28], [31], [32], [34], [35], [36], [37]) over the follow-up period. They have also resulted in higher reporting of safer injection practices such as needle disinfection [19], [20], [22], [24], [28], [34], [38] and increased enrollment in drug treatment programs [21], [22], [27], [30], [38], [39]. Other studies have also demonstrated a potential for PDIs to increase adherence to medical care for active IDUs [7] and street-based female sex workers with HIV [13].

In drug using populations, PDIs typically outperform traditional outreach interventions (TOIs) with respect to reductions in high-risk behaviors (e.g., dividing drugs by filling one syringe with drug solution and then expelling a portion into the hub (frontloading) or barrel (backloading) of a second syringe [17], crack cocaine use [40], injection frequency [5], [12], sharing of cookers/filters, rinse water, and syringes [5]) over follow-up. HIV prevention education has also been more effective in PDIs than in TOIs [5]. While PDIs and TOIs differ in both the way participants are recruited (by peers in PDIs and by outreach workers in TOIs) and in how the intervention is delivered (by peers in PDIs and by outreach workers in TOIs), most comparison studies attribute differences in the intervention outcome solely or primarily to the way in which the interventions are disseminated. However, because the control and intervention groups were not similarly recruited, some of these changes may also reflect differences in how individuals were recruited. In these studies, it is not possible to remove the effects due to peer-recruitment from the measured intervention effect. In other words, risk of behavior change resulting from increased interaction with peers among those who were recruited by peers but not among those recruited by outreach workers cannot be removed from the outcome measures in these studies.

Prior studies have shown that drug users recruited by outreach workers and RDS differ significantly on baseline characteristics [5], [41], [42], however studies have not examined whether the recruitment approach influences changes in these characteristics and behaviors over time. Given that RDS is currently being used to evaluate the effectiveness of peer-driven HIV prevention interventions prospectively and to recruit participants for longitudinal studies that assess changes in drug use over time [33], studies are needed to verify that changes in individual risk behaviors and health-seeking behaviors (e.g., drug treatment entry and HIV testing patterns) prospectively are not different for those recruited by peers and outreach workers in the absence of an intervention. Furthermore, findings from an ethnographic report by Scott suggest that RDS may alter the network composition of recruits and recruiters [43]. He proposed that recruitment incentives may encourage RDS recruiters to exercise their social influence on their peers and that the recruitment process may permit social interactions between drug users who did not previously know one another, thereby promoting the expansion of high-risk networks among research participants and consequently increasing HIV transmission [43]. Because changes in network composition that are induced by peer recruitment (i.e., increased interaction with peers that is related to the study design) could also introduce bias into the outcome measures in intervention evaluations, it is also necessary to evaluate prospective changes in social network composition among individuals recruited by their peers, but in the absence of an intervention.

Both of these points warrant an investigation of the potential biases that may be introduced in prospective studies that rely on peer recruitment. This analysis will determine whether RDS and targeted street outreach recruits (who do not receive an intervention) differ with respect to changes in health-seeking behaviors, namely utilization of HIV testing and drug treatment services, and network composition over a 6-month period of follow-up among an illicit drug using study population. Significant differences in behavior changes and network composition over a 6 month period of time among those recruited by peers and outreach workers would suggest that prospective measures may be biased. No differences in behavior changes and network composition over a 6 month period of time among those recruited by peers and outreach workers would provide evidence that the outcome measures resulting from intervention evaluations and prospective RDS studies are not biased.

## Methods

### Ethics statement

The following study procedures and documents have been reviewed and approved by the institutional review boards at Columbia University and the New York Academy of Medicine. All participants provided written informed consent to participate in this study.

The data for this analysis were collected as part of Social Ties Associated with Risk of Transition into injection drug use (START), a longitudinal study, which aimed to identify social risk factors for initiating injection drug use among young adult non-injection and newly initiated injection drug users (heroin, crack, and cocaine) in New York City. The methods of this study have been reported previously [41]. In brief, NIDUs and IDUs were recruited concurrently through targeted street outreach and RDS between July 2006 and June 2009. Non-injection drug users (NIDUs) were interviewed every 6 months for 18 months and newly initiated injection drug users (IDUs) completed a cross-sectional survey.

### Recruitment

As previously described, economically disadvantaged and racially diverse New York City communities with high rates of HIV infection and overdose mortality were ethnographically mapped and targeted [41]. Forty-six RDS seeds and all targeted street outreach participants were recruited concurrently using random street intercept sampling in these neighborhoods. Outreach recruitment followed a targeted sampling plan, which was developed for HIV prevention studies and has been used to recruit a convenience sample of those at increased risk for HIV [44], [45]. RDS participants received 3 coupons to recruit drug-using peers to participate in the study and both modes of recruitment were administratively ended in June 2009. Forty-six RDS seeds (28 of whom recruited eligible peers) and a maximum of 14 recruitment waves produced 357 peer-recruits. Two seeds, each extending ≥13 waves recruited over half the peer-recruits (n = 203). Five seeds (extending ≥6 waves each) recruited 255 individuals and 311 individuals were recruited by 10 seeds with recruitment waves extending ≥4 waves. 18 seeds did not recruit any eligible peers [41]. In total, 403 participants were recruited through RDS and 217 were recruited through targeted street outreach [41].

### Criteria for Eligibility

Eligible START participants were 18–40 years of age (verified with a photo ID) and active drug users. Eligible IDUs (N = 130) reported injecting heroin, crack or cocaine for 4 years or less and injecting at least once in the past 6 months. NIDUs (N = 490) reported non-injection use of heroin, crack or cocaine for at least one year and used heroin, crack or cocaine 2–3 times per week in the last three months (N = 490). Self-reported drug use was verified with a rapid drug test which screened for opiate and cocaine metabolites in the urine. The presence of metabolites validated drug use in the 2–3 days prior to the test. Those with a negative drug test were not eligible but were compensated for travel to and from the research site.

### Study Instrument

After providing written informed consent, all participants completed a 90 minute interviewer-administered baseline survey. NIDUs (but not IDUs) returned 6 months later to complete a follow-up questionnaire. Both surveys ascertained demographic and social contextual characteristics, information about his/her drug use, and network composition. However, baseline questionnaires collected information about one's network over the past year and the 6 month survey collected information about one's network in the past 6 months. Participants received $30 and round-trip transportation for completing each questionnaire.

### Additional Study Procedures

After completing the baseline survey, participants recruited through respondent driven sampling also received 1) three RDS coupons to recruit drug-using peers to participate in START, 2) an individual recruitment training with an interviewer to emphasize the importance of peer recruitment and provide tips on peer recruitment, and 3) an invitation to attend up to two group-facilitated peer recruitment training sessions (RDSTs) offered bi-weekly. RDS-recruited participants speaking only Spanish received an extended individual recruitment training since there were too few Spanish-speaking participants to conduct RDSTs in Spanish. Those attending RDSTs received $20 and round-trip transportation after completing a post-session survey that collected information about their experiences with peer recruitment and feedback regarding the session.

As neither group received an intervention, the samples recruited through RDS and targeted street outreach differed with respect to participant recruitment. Additionally, RDS participants (but not those recruited by targeted street outreach) received advice on how to recruit peers (e.g., individual recruitment trainings, group-facilitated peer recruitment training sessions, and/or extended individual recruitment trainings).

### Data analysis

This analysis was restricted to NIDUs (N = 490), as only NIDUs completed the 6 month follow-up survey. Of 490 NIDUs, 2 were removed from this analysis because of incomplete network information (one targeted street outreach recruit and one RDS recruit). An additional 159 participants were removed from this analysis because they did not complete the 6-month follow-up survey, for a final sample size of 329 (N = 92 TSO recruits and 237 RDS recruits).

### Outcome variables

#### Changes in HIV testing behaviors

It is recommended that injection drug users and heavy non-injection drug users seek testing for HIV every 6 months. Using this recommendation as a guideline for our analysis, we evaluated differences in self-reported HIV testing practices over the past 6 months at baseline and at the 6 month follow-up visit. Two variables were created to evaluate changes in HIV testing behavior and individuals who were HIV positive at baseline (N = 44) and who were missing information on HIV testing at either baseline or at the follow-up visit (N = 13) were excluded. To evaluate increases in HIV testing behavior, a variable was created to compare those who reported a recent HIV test at the 6-month follow-up visit (an HIV test between the baseline and 6-month follow-up visit) but no HIV test in the 6 months prior to the baseline interview (N = 50) to those who did not report a recent HIV test at either study visit (N = 25). To evaluate decreases in HIV testing behavior, a variable was created to compare those who reported receiving an HIV test 6 months prior to the baseline survey but not between the baseline and 6-month follow-up survey (N = 25) to those who reported a recent HIV test at both study visits (N = 170). One individual who reported an HIV test confirming his/her HIV positive status in the 6 months prior to the baseline survey was excluded from the variable comparing those who reported receiving an HIV test 6 months prior to the baseline survey but not between the baseline and 6-month follow-up survey to those who reported a recent HIV test at both study visits. Thus, 270 individuals were used to create these two variables; 75 individuals were used to assess increases in HIV testing behavior and 195 individuals were used to assess decreases in HIV testing behavior.

#### Changes in drug treatment utilization

Two variables ascertained changes in drug treatment utilization. To evaluate decreases in drug treatment utilization, individuals who reported utilizing any form of drug treatment in the 6 months prior to the baseline survey but not in the time between the baseline and 6-month survey (N = 48) were compared to those who reported utilization of any form of drug treatment in the 6 months prior to each study visit (N = 84). To evaluate increases in drug treatment utilization, individuals who reported utilizing any form of drug treatment between the baseline and 6-month survey visit but not in the 6 months prior to the baseline (N = 63) were compared to those who did not report utilizing any form of drug treatment in the 6 months prior to either study visit (N = 134).

#### Changes in network composition

At baseline, participants were asked to list the names, nicknames, or initials for each person in the past year 1) whom he/she could borrow $25 from, 2) who would let him/her stay at their place, 3) who he/she could talk to about personal or private matters, 4) who he/she used drugs with, 5) who he/she had sex with, 6) who he/she could ask for advice about health care or medical service, 7) who he/she could talk to about issues related to drug use (e.g., how to use drugs safely) and 8) who he/she could get information about social services like housing, welfare or social security. Individuals who were listed in 6–8 above were combined to create a variable to represent informational social support networks.

The number of unique individuals recorded was his/her total network size. Participants were then asked to provide information about each of the names provided (i.e., demographic characteristics, history of incarceration, and information about whether he/she injected drugs, smoked crack, or snorted heroin). The proportion of drug using sex, incarcerated and social support networks at each study visit was calculated using the total network size at that study visit as the denominator. Network proportions at baseline were subtracted from network proportions at the 6-month follow up to evaluate changes in network composition over the study period.

### Data analysis

Descriptive statistics were used to characterize the sample. Chi-square statistics were used to compare RDS- and targeted street outreach-recruited participants with respect to changes in HIV testing behaviors and drug treatment utilization over the past 6 months. T-tests were used to compare RDS- and targeted street outreach participants with respect to changes in his/her network composition over the past 6 months. As there were no major differences in homophily or drug using network size by any variables considered (e.g., gender, race/ethnicity, education, income, age, homelessness in the past 6 months, injection status, HIV status, heroin use in the past 6 months, cocaine use in the past 6 months, and crack use in the past 6 months) and the weights corresponding with each of these characteristics were low, weighted and unweighted RDS estimates did not differ significantly [41]. Because weighting one comparison group (RDS) and not the other (targeted street outreach) to correct for sampling biases could introduce additional biases to the comparison of these two sampling approaches, we did not apply weights to the respondent driven sample.

## Results

Of the 329 study participants (Table 1), the median age at baseline was 35 years (Interquartile range [IQR]: 30–38) and the median network size at baseline was 3 (IQR: 2–4). The sample was 66% male, 23% Hispanic, 69% black, 14% had a total annual income greater than $10,000, and 52% had graduated from high school or the equivalent. In the past 6 months, 62% reported being homeless, 86% used crack, 70% used cocaine, and 40% used heroin (Table 1). At baseline, 14% were HIV positive, 60% had been tested for HIV in the past 6 months, and 40% were enrolled in some form of drug treatment in the past 6 months. Of the 196 who reported an HIV test in the 6 months prior to the baseline survey, one reported testing positive for HIV (Table 1).

**Table 1:** Baseline demographics for NIDUs who completed both the baseline survey and the 6-month follow-up survey, NYC (2006–2009) N = 329.

Variable	N	%
Age, Median (IQR)	35	(30–38)
Total number of people in one's network in the past year, Median (IQR)	3	(2–4)
Total number of people in one's network in the past year, Mean (SD)	3.45	(2.66)
Used Crack (with or without heroin)	283	86.0
Used Cocaine (with or without heroin)	228	69.5
Used Heroin (with or without crack/cocaine)	130	39.8
HIV positive	44	14.2
Homeless in the past 6 months	204	62.0
Total annual income >$10,000	43	13.9
Education≥High school	170	51.7
Hispanic	77	23.4
Black	226	68.7
White/Other	26	7.9
Male	215	65.8
RDS Recruit	237	72.0
Targeted Street Outreach Recruit	92	28.0
Enrolled in drug treatment in the past 6 months	132	40.1
Received an HIV test in the past 6 months	196	59.6

Most participants listed 0 or 1 network for each network category (Table 2). At baseline there were no significant differences in the total number of networks reported (p = 0.19) or in the number of drug using networks reported (p = 0.06) between those recruited with targeted street outreach and RDS. Over the 6 month follow-up period, there were no significant differences between those recruited with targeted street outreach and RDS with respect to changes in network composition (Table 3). On average, participants reported more networks at the 6 month assessment than they did at baseline (median change = 1.0; IQR: −1, 2). This pattern was observed among those recruited by both targeted street outreach and RDS. Overall, the median change in the network composition for each of the characteristics assessed between baseline and follow-up was zero and this difference was not significantly different by recruitment strategy (Table 3).

**Table 2:** Baseline network characteristics of NIDUs who completed both the baseline survey and the 6-month follow-up survey, NYC (2006–2009) N = 329.

	In the past year, the number of people in your network who _____			Proportion of total networks in the past year who _____		
Variable	Median	IQR		Median	IQR	
You could borrow $25 from	1	0	1	0.33	0	0.50
Would let you stay at their place	1	0	1	0.25	0	0.50
You could talk to about personal or private matters	1	0	1	0.33	0.13	0.50
You used drugs with	1	0	2	0.33	0	0.53
You had sex with	1	1	1	0.50	0.25	0.67
You could ask for advice about health care/medical services	1	0	1	0.17	0	0.50
You could talk to about issues related to drug use	0	0	1	0.14	0	0.33
You could get information about social services from	0	0	1	0.12	0	0.33
Provided informational support	1	0	1	0.33	0	0.50
Sniffed heroin	0	0	0	0	0	0.11
Smoked crack	1	0	2	0.33	0	0.62
Injected	0	0	0	0	0	0
Used drugs	1	0	2	0.50	0.17	0.73
Paid/were paid for sex	0	0	1	0	0	0.25
Were in jail	0	0	1	0	0	0.33

**Table 3:** Comparison of RDS and TSO-recruited participants with respect to changes in network composition over 6 months.

	ALL (N = 329)	TSO (N = 92)	RDS (N = 237)	
Change in proportion of networks who ______	Median (IQR)	Median (IQR)	Median (IQR)	P-Value
You could get information about social services from	0 (−0.25, 0.17)	0 (−0.23, 0.09)	0 (−0.30, 0.17)	0.855
You could talk to about issues related to drug use	0 (−0.20, 0.17)	0 (−0.20, 0.07)	0 (−0.20, 0.20)	0.135
You could ask for advice about health care or medical services	0 (−0.25, 0.14)	0 (−0.23, 0.06)	0 (−0.25, 0.17)	0.152
Provided informational support (health/medical, drug issues, social services)	0 (−0.33, 0.33)	0 (−0.27, 0.20)	0 (−0.33, 0.33)	0.277
You had sex with	0 (−0.27, 0.17)	0 (−0.25, 0.17)	0 (−0.27, 0.17)	0.916
You used drugs with	0 (−0.33, 0.15)	0 (−0.33, 0.18)	0 (−0.33, 0.14)	0.911
You could talk to about personal or private matters	0 (−0.25, 0.25)	−0.04 (−0.32, 0.23)	0 (−0.25, 0.25)	0.219
Would let you stay at their house	0 (−0.25, 0.20)	0 (−0.28, 0.17)	0 (−0.25, 0.20)	0.338
You could borrow $25 from	0 (−0.25, 0.20)	0 (−0.21, 0.17)	0 (−0.25, 0.25)	0.571
Injected	0 (0, 0)	0 (0, 0)	0 (0, 0)	0.848
Smoked crack	0 (−0.33, 0.07)	0 (−0.33, 0.20)	0 (−0.38, 0)	0.242
Sniffed heroin	0 (0, 0)	0 (0, 0)	0 (0, 0)	0.261
Were in jail	0 (−0.17, 0)	0 (−0.09, 0)	0 (−0.20, 0)	0.413
Used drugs	0 (−0.33, 0.33)	0 (−0.29, 0.32)	0 (−0.33, 0.33)	0.821
Overall, change in number of networks over the past year	1 (−1, 2)	1 (−1, 2)	1 (−1, 2)	0.744

There were similarly no significant differences in HIV testing or drug treatment service utilization by recruitment strategy (Table 4). Of the 132 who reported recent enrollment in a drug treatment program at baseline, 36% did not report recent enrollment in a drug treatment program at the follow-up visit; there were no significant differences by recruitment strategy (RDS = 36% TSO = 38%; p = 0.837). Of the 197 who did not report recent enrollment in a drug treatment program at baseline, 32% reported recent enrollment in a drug treatment program at the 6-month follow-up visit; there were no significant differences by recruitment strategy (RDS = 33% TSO = 30%; p = 0.694). Nearly 67% of those who were HIV negative and who did not report a recent HIV test at baseline reported receiving an HIV test in the 6 months prior to the follow-up survey and there were no significant differences by recruitment strategy (RDS = 67% TSO = 65%; p = 0.854). Finally, of those who were HIV negative and who reported a recent HIV test at baseline, 87% reported a recent HIV test at their follow-up visit. Again, there were no significant differences by recruitment strategy (RDS = 85% TSO = 92%; p = 0.218).

**Table 4:** Comparison of RDS and TSO-recruited participants with respect to changes in drug treatment and HIV testing service utilization over 6 months.

		ALL (N = 329)	TSO (N = 92)	RDS (N = 237)	
Variable	Variable Categories	N (%)	N (%)	N (%)	P-Value
Increases in drug treatment utilization	No recent drug treatment enrollment at baseline and recent drug treatment enrollment at 6-month follow-up	63 (32.0)	13 (29.6)	50 (32.7)	0.694
	No recent drug treatment enrollment at baseline or at 6-month follow-up	134 (68.0)	31 (70.5)	103 (67.3)	
Decreases in drug treatment utilization	Recent drug treatment enrollment at baseline and no recent drug treatment enrollment at 6-month follow-up	48 (36.4)	18 (37.5)	30 (35.7)	0.837
	Recent drug treatment enrollment at baseline and at 6-month follow-up	84 (63.6)	30 (62.5)	54 (64.3)	
Increases in HIV testing behavior	No recent HIV test at baseline and recent HIV test at 6-month follow-up	50 (66.7)	13 (65.0)	37 (67.3)	0.854
	No recent HIV test at baseline or at 6-month follow-up	25 (33.3)	7 (35.0)	18 (32.7)	
Decreases in HIV testing behavior	Recent HIV test at baseline and no recent HIV test at 6-month follow-up	25 (12.8)	5 (8.3)	20 (14.7)	0.218
	Recent HIV test at baseline and at 6-month follow-up	171 (87.2)	55 (91.7)	116 (85.3)	

### Baseline Differences

While there were no significant changes in network composition, or HIV testing and/or drug treatment service utilization over the 6 month period by recruitment strategy, there were significant differences between these groups at baseline (Tables 5 and 6). For example, individuals recruited with RDS were significantly less likely to have recently been enrolled in a drug treatment program at baseline (36% compared with 48%, respectively; p = 0.0181; Table 5). At baseline, participants recruited by targeted street outreach reported a significantly greater proportion of networks who would let him/her stay at their house (p = 0.0153) and who he/she could talk to about personal or private matters (p = 0.0043) and a significantly smaller proportion of heroin sniffing networks than those recruited with respondent driven sampling (p = 0.0487) (Table 6). Regression models were fit to account for baseline differences by recruitment strategy. After adjustment, changes in recent HIV testing behaviors, drug treatment enrollment, and network composition between the baseline and 6 month follow-up survey did not differ by recruitment approach (data not presented).

**Table 5:** Baseline differences in recent HIV testing and recent drug treatment enrollment by recruitment strategy.

	TSO (N = 124)	RDS (N = 365)	
Variable	N (%)	N (%)	P-value
Enrolled in drug treatment in the past 6 months	59 (47.6)	130 (35.6)	0.0181
HIV test in the past 6 months	78 (72.9)	218 (73.7)	0.5536

**Table 6:** Baseline differences in network composition by recruitment strategy.

	TSO (N = 124)	RDS (N = 365)	
Change in the proportion of networks in the past year who ______________	Median (IQR)	Median (IQR)	P-value
You could borrow $25 from	0.33 (0.06, 0.63)	0.33 (0.00, 0.50)	0.0845
Would let you stay at their house	0.33 (0.00, 0.50)	0.20 (0.00, 0.50)	0.0153
You could talk to about personal or private matters	0.33 (0.23, 0.67)	0.33 (0.00, 0.50)	0.0043
You used drugs with	0.33 (0.00, 0.58)	0.33 (0.00, 0.56)	0.9704
You had sex with	0.40 (0.25, 0.73)	0.50 (0.25, 0.67)	0.7800
You could ask for advice about health care or medical services	0.20 (0.00, 0.50)	0.13 (0.00, 0.38)	0.1142
You could talk to about issues related to drug use	0.20 (0.00, 0.42)	0.00 (0.00, 0.33)	0.0839
You could get information about social services from	0.14 (0.00, 0.33)	0.00 (0.00, 0.33)	0.5727
Provided informational support (health/medical, drug issues, social services)	0.33 (0.00, 0.67)	0.25 (0.00, 0.50)	0.0586
Sniffed heroin	0.00 (0.00, 0.33)	0.00 (0.00, 0.13)	0.0487
Smoked crack	0.20 (0.00, 0.50)	0.33 (0.00, 0.67)	0.0806
Injected	0.00 (0.00, 0.00)	0.00 (0.00, 0.00)	0.6558
Used drugs	0.50 (0.00, 0.69)	0.50 (0.20, 0.75)	0.5371
Paid/were paid for sex	0.00 (0.00, 0.25)	0.00 (0.00, 0.33)	0.8101
Were in jail	0.00 (0.00, 0.29)	0.00 (0.00, 0.33)	0.4282

## Discussion

These findings provide no evidence to suggest that changes in network composition or health seeking behaviors (e.g., HIV testing or drug treatment utilization) over time differ by recruitment approach. Contrary to qualitative findings by Scott, individuals recruited with RDS were no more likely than those recruited with targeted street outreach to form social ties with higher-risk individuals [43]. Our findings are more generalizable and represent a less biased sample than those from Scott's qualitative report, as the sample selected by Scott was highly selective in that it was comprised of only those individuals enrolled in Chicago's HIV behavior surveillance study among IDUs who had successfully recruited the maximum number of peer networks. Of the 529 IDUs enrolled in the parent study, 70 were eligible to participate in Scott's sub-study, and the sample was further restricted to the 25 individuals considered to be the most active members of the coupon economy. In addition, changes in the self-reported use of HIV testing or drug treatment services over the 6 month period of follow up did not differ by recruitment approach, which supports prior findings from prospective HIV behavioral surveillance and intervention studies that compare peer driven interventions with traditional outreach interventions.

### Limitations

There are several limitations to this analysis. First, no change in network composition from baseline to follow-up was indicated as a “zero” change. Thus, individuals who had low risk networks both at baseline and at follow-up were grouped together with those who had high risk networks at both baseline and at follow-up. Similarly, a reduction of 50% for a person with 10 networks may be qualitatively different from a similar reduction in a person with 2 networks. However, even after controlling for the total network size at baseline (in a separate analysis), there were no significant differences. Additionally, we were not able to ascertain whether or not the network members reported in the baseline were the same or different individuals from those reported in the 6 month follow-up survey or vice versa. Therefore, a change in the composition of one's network might reflect a change in the roles or characteristics of the same network members reported at baseline or a change in the individuals making up one's network. Similarly, no change in the composition of networks may reflect the fact that the same individuals are reported in the baseline and follow-up interviews, or that different individuals with similar characteristics or roles are reported in each survey. However, the influence of one's social environment on individual risk behaviors has been extensively studied in drug using populations [46], [47], [48], [49], [50], [51], [52], [53] and the proportion of high risk networks has been previously identified as a social factor associated with HIV risk behavior [49], [51], [54], [55] and is consequently an appropriate variable to assess one's social risk environment.

It is also possible that participants in this study under-reported their number of networks. Fewer drug-using networks and total networks were reported in this study than in other studies among NIDUs [53] and IDUs [56], [57]. For example, Weeks and colleagues [58] reported an average of 4.5 drug using peers [58] and Latkin and colleagues [57] reported an average of 5.22 drug using social networks [57], both of which are larger than the 1.58 drug using networks reported here. Each study also reported more total network members (including non-drug using networks; means: 5.6 [58] and 10.3 [57]) than what is reported here (mean = 3.45). Some of these differences may reflect the fact that this analysis was restricted to NIDUs, while the studies conducted by Weeks and colleagues [58] and Latkin and colleagues [57] were among IDUs. However, a study among NIDUs conducted by Pilowsky and colleagues [53] reported a median total network size of 5 and a median drug using network size of 2 [53], which is still higher than what we report. However, because this analysis evaluated changes in the proportion of networks reported rather than the absolute number of networks reported, this bias is likely to be minimal. In addition, individuals were asked to report characteristics about each network member and it is not known how well self-reported network characteristics approximate actual network characteristics. HIV testing behaviors and drug treatment utilization measures are also based on self-report so there is a potential for bias due to social desirability. However, this can also be considered a “methodological” strength, as the results suggest that RDS does not influence social desirability of reporting HIV testing and drug treatment enrollment. Additionally, networks reported at baseline were past-year networks, while those reported at follow-up referred to the past 6 months. Since we were assessing changes in network composition over a six month period, this bias is considered minimal. Also, as only NIDUs were followed prospectively, these findings can only be generalized to NIDUs. As most intervention studies are conducted among IDUs, similar findings should be replicated in an IDU sample. Similarly, replicate findings should also be produced among samples of men who have sex with men, sex workers, and other populations which commonly use RDS to sample from the target population.

This study had 80% power to detect an effect size of 1.8 or greater in the absence of a design effect. As such, we may be underpowered to detect significant differences between groups recruited with RDS and targeted street outreach. However, a ‘zero’ change was observed with respect to each of 14 network composition variables that share the same denominator. Using a more conservative approach that accounts for multiple comparisons with a Bonferroni correction would have set the significance level for the family of tests (change in network composition) to be 0.05. As each of the 14 network composition variables share the same denominator, the Bonferroni correction would test each of the individual tests at a significance level of 0.05/14 (α = 0.004). This approach, which evaluates “change in network composition” as a family of tests is more conservative and provides additional support for our conclusion of no significant change in network composition over the 6 month period. At the very least, our analysis suggests that peer recruitment alone is not likely to alter network structure over a 6 month period. However, these findings should be replicated in a larger sample with the power to detect minute changes in network composition and health-seeking behaviors.

Finally, of the 489 NIDUs interviewed at baseline, only 329 (67%) returned for the 6-month follow-up survey. As retention was moderate, we compared individuals who were and were not retained with respect to the variables of interest in this analysis. Retention was lower for respondent-driven recruits (65%) than for targeted street outreach recruits (74%), but this difference was not statistically significant (p = 0.06). Compared with NIDUs who completed both surveys, NIDUs who were loss to follow-up were significantly younger, reported using drugs with fewer people, reported that a greater proportion of their network sniffed/snorted heroin, were more likely to use cocaine and heroin, to be homeless in the past 6 months, to be HIV negative, and to be Hispanic but these factors did not differ by recruitment approach.

While there is some evidence to suggest differential loss to follow-up by age, race/ethnicity, homelessness, HIV status, drug use, and the self-reported number of people whom he/she uses drugs with, these differences did not vary by mode of recruitment. Consequently, it is likely that we would have observed greater reductions in the proportion of networks who individuals could rely on for informational support, who sniffed or snorted heroin, who injected drugs, and who they had sex with over the follow-up period had all of the participants interviewed at baseline completed both interviews, however these reductions would be similar among those recruited with RDS and targeted street outreach.

### Strengths

There are also several strengths to this analysis. While RDS was designed as a tool for sampling and inference in cross-sectional samples, it has been more recently adopted to measure changes in HIV-risk behaviors and drug use prospectively. However, the appropriateness of using RDS for prospective analyses has never been evaluated. In addition, it is generally accepted that PDIs outperform TOIs, however studies comparing the two approaches have not isolated true intervention effects because the comparison samples differ in the way that individuals were recruited and in how the interventions were delivered. This study compares these two approaches in the absence of an intervention, consequently isolating the effect of recruitment strategy. Finally, the 6 month time frame used in this study is appropriate, as it is comparable to the follow-up period used in many behavioral intervention studies.

### Conclusion

With limitations acknowledged, this prospective study provides strong evidence supporting findings from prior studies that have compared PDIs and TOIs. As this study found no significant differences in the changes in health-seeking behaviors or in the social network composition over the 6 month follow-up period by recruitment approach, it is likely that the changes observed in PDIs can be attributed to differences in how the intervention was delivered and not to differences related to the manner in which participants were recruited. Further, the findings from this study suggest that those recruited via RDS were no more likely than those recruited by targeted street outreach to form riskier networks as a consequence of their participation in a research study using peer-driven recruitment. These findings should be replicated in studies with larger sample sizes and with different target populations to further support the use of RDS in prospective studies. Finally, although RDS was not specifically designed as a tool for prospective analyses, these data suggest that RDS may be an appropriate analytic tool for measuring prospective changes in HIV risk behavior and HIV incidence.
